# Annotations

**Published:** 1898-05-21

**Authors:** 


### ANNOTATIONS.

### War and Disease.

IT is becoming very apparent that if it should be necessary for the United States to invade Cuba and occupy that country in force, the war will have to be another example of what have come to be spoken of as "doctor's wars," expeditions which depend for their success on the perfection of their medical and sanitary departments. Fighting power is good, but final victory will come to those who can keep their fighting men alive; and, unfortunately for the Spaniards, that is where they seem to have failed disastrously. The New York *Medical News* publishes extracts from a report drawn up by Dr. Brunner, who was last year sent to Cuba as the representative of the United States Marine Hospital Service to keep them informed of the prevalence of infectious diseases there. He resided there from April, 1897, to April, 1898, and his report shows what enormous losses the Spanish troops have suffered from disease alone, even in the more civilised parts of the island. During the year 1897 the deaths from yellow fever in the military at Havana and Regla amounted to 2,583. During the same time 3,451 deaths from that disease occurred among the soldiers in other parts, so that the total number of deaths from yellow fever occurring in military hospitals in 1897 was no less than 6,034, which would represent about 30,000 cases of yellow fever in the year among the troops. The deaths from typhoid fever are estimated at 2,500, those from malarial fevers at 7,000, and those from enteritis and dysentery at no less than 12,000. In regard to enteritis and dysentery nearly 5,000 deaths are known to have occurred in the five military hospitals in Havana alone, and in these hospitals the patients were better fed and cared for than at any other points on the island. Summing up to the matter we have the following figures, which are given as approximately correct, for the army of occupation during the year 1897: Deaths from yellow fever, 6,034; deaths from enteric fever, 2,500; enteritis and dysentery, 12,000; malarial fevers, 7,000; all other diseases, 5,000; total, 32,534. This gives one some idea of what is meant when Cuba is spoken of as draining away the manhood of Spain. Small need, one would think, to fight an army which is so ill-cared for that it dissolves away at such a rate. A blockade should be sufficient.

### Pleuro-pneumonia.

THE annual report of the Chief Veterinary Officer to the Board of Agriculture which has just been presented to Parliament contains an admirable *résumé* of what is at present known about that most important disease pleuro-pneumonia, a disease which, but for the constant watchfulness of the officers of the Board, would certainly spread with disastrous effect among our cattle. The study of epidemics among the lower animals is most important, and in careful hands becomes even more prolific of results than similar investigations among men can ever be; for, by the slaughter of suspected animals, it is possible to settle many problems which in regard to men must always remain more or less matters of guess-work. Nothing is more curious in the history of epidemics than the manner in which certain individuals appear to walk unharmed through the infection which surrounds them on every side, and to expose themselves without evil consequences to dangers to which others succumb helplessly and at once. Few things, again, are more puzzling than the way in which an epidemic sometimes appears to burst into being apparently without the slightest trace of connection with any other similar outbreak, notwithstanding that in the developed epidemic it is clear that the malady spreads by infection from case to case. The same thing is found in regard to pleuro-pneumonia in cattle, and the explanation is full of interest. It has often been observed, when pleuro-pneumonia breaks out in a herd, and all the apparently diseased beasts have been slaughtered or removed, that a certain number of those remaining will live through the outbreak, continuing, perhaps, for months, without presenting obvious symptoms of the disease; and it has been thought that they have escaped infection by virtue of some peculiar form of immunity which they possessed. Since, however, whole herds, consisting sometimes of 100 or more head of cattle, have been slaughtered on the occurrence of pleuro-pneumonia among them, it has been found that these immune cattle have not really resisted the disease, but are immune by virtue of having already had it. They are, in fact, the original sinners who have introduced the disease into the herd; they have already had pleuro-pneumonia in a localised form, and recovered without exciting any suspicion of illness in the mind of the attendant. Here we have the story of diphtheria over again, and no doubt it is the story of many an infectious disease—the mild case, perhaps the long series of mild cases, unnoticed by everyone, spreading infection right and left, while remaining apparently immune throughout the epidemic. But pleuro-pneumonia leaves its mark within the lungs, and, on slaughter of the herd, the whole history of the outbreak lies bare before us.

### Isolation after Measles.

IN a paper read before the Society of Medical Officers of Health on the sanitary supervision of schools, Dr. Reginald Dudfield said that the rule he had followed in regard to disinfecting after measles was to cause a visit to be made to the house at the end of the third week after the date of the onset of the first reported case. If no case had occurred within the fortnight immediately preceding the second visit, the disinfection was carried out, and the family declared free from infection. If secondary cases had occurred, a further period of three weeks was reckoned from the day of sickening of the last case, at the end of which a further visit was made, and so on until the place was disinfected. So far so good. But what are we to think of the next suggestion, which is as follows: "I think it should be made law that no children belonging to a family infected with measles should be allowed outside the house or tenement until the family has been declared free from infection"? Poor little children! Those who know the condition of London tenement life may well pity the lot of the youngsters whom it is proposed thus to shut up week after week in the crowded rooms which they call "home." The society, however, was by no means unanimous as to the practicability or even necessity of disinfection of all houses after measles.

